# Reconciliation of Genome-Scale Metabolic Reconstructions for Comparative Systems Analysis

**DOI:** 10.1371/journal.pcbi.1001116

**Published:** 2011-03-31

**Authors:** Matthew A. Oberhardt, Jacek Puchałka, Vítor A. P. Martins dos Santos, Jason A. Papin

**Affiliations:** 1 Department of Biomedical Engineering, University of Virginia, Charlottesville, Virginia, United States of America; 2 Helmholtz Center for Infection Research (HZI), Braunschweig, Germany; 3 Laboratory of Systems & Synthetic Biology, Wageningen University, Wageningen, The Netherlands; University of California San Diego, United States of America

## Abstract

In the past decade, over 50 genome-scale metabolic reconstructions have been built for a variety of single- and multi- cellular organisms. These reconstructions have enabled a host of computational methods to be leveraged for systems-analysis of metabolism, leading to greater understanding of observed phenotypes. These methods have been sparsely applied to comparisons between multiple organisms, however, due mainly to the existence of differences between reconstructions that are inherited from the respective reconstruction processes of the organisms to be compared. To circumvent this obstacle, we developed a novel process, termed metabolic network reconciliation, whereby non-biological differences are removed from genome-scale reconstructions while keeping the reconstructions as true as possible to the underlying biological data on which they are based. This process was applied to two organisms of great importance to disease and biotechnological applications, *Pseudomonas aeruginosa* and *Pseudomonas putida*, respectively. The result is a pair of revised genome-scale reconstructions for these organisms that can be analyzed at a systems level with confidence that differences are indicative of true biological differences (to the degree that is currently known), rather than artifacts of the reconstruction process. The reconstructions were re-validated with various experimental data after reconciliation. With the reconciled and validated reconstructions, we performed a genome-wide comparison of metabolic flexibility between *P. aeruginosa* and *P. putida* that generated significant new insight into the underlying biology of these important organisms. Through this work, we provide a novel methodology for reconciling models, present new genome-scale reconstructions of *P. aeruginosa* and *P. putida* that can be directly compared at a network level, and perform a network-wide comparison of the two species. These reconstructions provide fresh insights into the metabolic similarities and differences between these important *Pseudomonads*, and pave the way towards full comparative analysis of genome-scale metabolic reconstructions of multiple species.

## Author Summary

Over the past decade, the increasing availability of fully sequenced genomes, functional genomics databases, and a broad existing scientific literature on metabolism have been leveraged towards the generation of genome-scale metabolic reconstructions for a wide variety of organisms. A major hurdle in the field, however, is the lack of standardization in this ‘metabolic reconstruction’ process, which makes it difficult to compare reconstructions of multiple species with each other. Notably, it is difficult to determine if differences seen between the models are of biological origin, or if they reflect noise from the respective reconstruction processes. To address this problem, we have developed a novel method, termed metabolic network reconciliation, in which genome-scale models of two well-studied and phylogenetically related bacteria (*P. aeruginosa* and *P. putida*) were compared such that differences between the models not upheld by biological evidence were eliminated. These reconciled models were re-validated with experimental data, and used to explore differences in the biology of these organisms and to gain insight into the reconstruction process itself.

## Introduction

With the development of rapid genome sequencing methodologies and powerful, scalable computational tools, the past decade has seen the generation of an increasing number of genome-scale metabolic reconstructions (metabolic GENREs) [1]. These reconstructions generally account for the functions of hundreds to thousands of genes, and are intended to incorporate all known metabolic reactions for a particular organism into a standardized format, enabling the generation of a computational model that can be analyzed with a variety of emerging mathematical techniques [2].

Despite the reconstruction of over 50 metabolic GENREs to date, little effort has been put towards comparison of multiple species at a genome level with a network-centric approach. Such a comparison is bound to yield interesting insights into the relationships between the structure of a metabolic network and the resulting phenotype of an organism, as well as contribute to the explanation of various physiological features such as virulence pathways and unique metabolic capabilities. Yet in order to draw meaningful conclusions from such a comparison, it is necessary to ensure that the identified differences are representative of true differences between the organisms, rather than artifacts from the reconstruction processes.

The metabolic reconstruction process integrates the genome sequence and annotation of an organism with a multitude of different sources, including biological databases (e.g. Expasy, KEGG, BRENDA) and primary literature, to construct the metabolic GENRE [3]. A key difficulty in the reconstruction process is that these sources can contain incomplete and contradictory information, including ‘putative’ and ‘probable’ gene annotations, descriptions of enzymatic functions that require interpretation in order to be linked to specific substrates (e.g., substrates such as ‘acceptor’ and ‘long-chain-acyl-CoA’), vague or missing data about reaction reversibility, and varying or often unknown enzyme efficiencies. Due to these issues, building a metabolic GENRE involves hundreds of decisions as to which genes possess which function, which reactions should be included, and finally in which direction these reactions occur. As these decisions are often based on ambiguous and even conflicting data, there is a high risk that, when two independently created GENREs are compared, a considerable number of the differences observed would be caused by the ‘noise’ in the reconstruction process itself rather than representing actual biology. Therefore, making any informative conclusions from a comparison between metabolic GENREs necessitates a prior preprocessing that brings the reconstructions to a common standard pertaining to naming conventions and, more importantly, that identifies whether the observed differences between the reconstructions are upheld by the biological evidence.

To address these concerns, we have performed the first metabolic GENRE reconciliation, a process of eliminating erroneous differences between two existing metabolic GENRE**s**. Reconciliation is similar to but distinct from consensus building efforts such as metabolic reconstruction jamborees [4], as the focus is specifically on aligning metabolic reconstructions of two organisms to eliminate unverifiable differences, rather than merging different data to improve the metabolic GENRE of a single organism. This process was performed for the related but phenotypically distinct species, *Pseudomonas aeruginosa* (PAO) and *Pseudomonas putida* (PPU). These bacteria represent an ideal pair of organisms for a genome-scale metabolic comparison due to their tremendous scientific and medical importance. *P. aeruginosa* is an opportunistic pathogen, notorious for its chronic inhabitance of the lungs of cystic fibrosis patient and its role in causing acute and deadly nosocomial infections in immunocompromised patients [5],[6],[7]. Both *P. aeruginosa* and *P. putida* are ubiquitous environmental organisms, capable of living varied lifestyles in many habitats, and are of interest for biotechnological applications [8], [9], [10], [11], [12]. However, unlike *P. aeruginosa*, *P. putida* is not a human pathogen. This last feature makes reconciliation of these two important species particularly valuable, as it paves the way for comparative analyses that could lend insight into metabolic features contributing to pathogenicity.

The metabolic GENRE of each of these species was previously published by our groups [13], [14], and each reconstruction accounts for the metabolic function of approximately 1000 genes, along with gene-protein-reaction associations and full stoichiometric representations of the majority of known metabolic reactions present in the genome of each bacterium. As a result of the reconciliation process we describe in this paper, we have developed new reconstructions of *P. aeruginosa* and *P. putida*, labeled iMO1086 and iJP962 respectively following standard conventions [15]. These reconciled GENREs, like the initial GENREs, have been validated with BIOLOG substrate utilization data and viability data for transposon-derived mutants. The reconstructions achieve similar degrees of accuracy as the original reconstructions they were based on, and as a result of the elimination of non-biological differences between the metabolic networks, are now amenable to functional comparison between species. We use these reconciled models to perform the first genome-scale comparison of metabolic flexibility of *P. aeruginosa* and *P. putida*, a comparison uniquely enabled by this reconciliation process, and to provide insight into metabolic factors that might help in characterizing the lifestyle capabilities among these bacteria.

## Results

**Figure 1:**
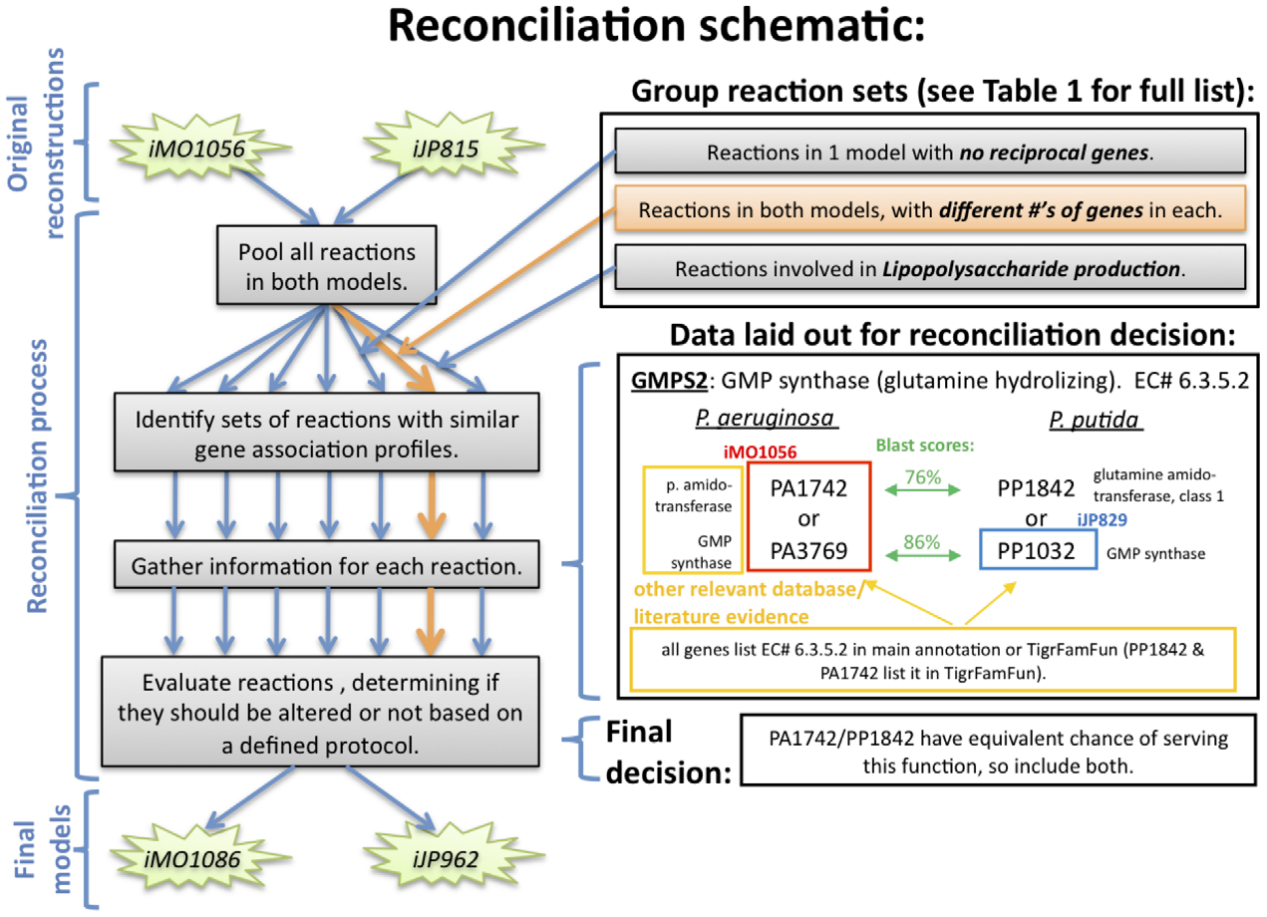
Format of reconciliation process. The reconciliation information for the reaction GMP synthase is shown. In the center are boxes showing the genes included in the original reconstructions, iMO1056 (*P. aeruginosa*) and iJP815 (*P. putida*). Reciprocal gene matches based on the genome-wide BLAST study are shown in the center with two-headed arrows. Auxiliary information about the genes is displayed on the left and right margins. Finally, the bottom box shows the final decision that was made for this reaction, based on the available information.

Network reconciliation is a process in which two previously reconstructed metabolic GENREs are compared with the null hypothesis that given no discriminating biological evidence for a given reaction to be included in one reconstruction but not the other, the reactions should be identically included in both. If sufficient evidence emerges that there is in fact a difference between the two organisms, then those differences are preserved in the final ‘reconciled’ GENREs that emerge from the process. The process of reconciliation is detailed in **Figure 1** with a specific example from *P. aeruginosa* and *P. putida*, and different components of the process are expanded in the upcoming sections.

The result of this process is a pair of metabolic GENREs for which every difference has been examined and verified with genomic, physiological, or literature-derived evidence. Finally, the two reconciled GENREs are grown *in silico* via Flux Balance Analysis (FBA) [16] and the ability of the final reconstructions to produce biomass is ensured. These fully functional genome-scale metabolic reconstructions can then be compared against one another in confidence that differences reflect biological differences in metabolism, as differences arising from reconstruction-based noise have been removed.

### Genome-scale homology search

As each metabolic reconstruction is a reflection of the genetic content of the respective organism, the comparison of two reconstructions requires identification of the overlap between genomic content of the compared organisms. As *P. putida* and *P. aeruginosa* are closely related genomically and phylogenetically, it was expected that many orthologs would exist with high homology, and thus shared functions. Therefore, as a first step in the reconciliation process, we identified highly homologous gene pairs, which we termed “reciprocal genes”. These reciprocal gene pairs were used in the reconciliation process to determine the similarity of gene associations for reactions in the two reconstructions, so identifying these pairs was a crucial first step for performing the reconciliation.

In order to identify reciprocal genes, we performed a homology search using the BLAST algorithm against the *P. putida* genome sequence database with *P. aeruginosa* genes as queries and vice versa. The searches were performed both with nucleotide and protein sequences. Two genes were termed reciprocal only if, in every aforementioned BLAST search performed with one of these genes as the query sequence, the other gene was reported as the first hit (e.g., *P. aeruginosa* gene X best matches *P. putida* gene X in protein and nucleotide BLAST, and vice versa). The metric used for comparing BLAST matches was the score of the highest scoring pair [17]. With this approach, a single gene can have at most one reciprocal gene. Altogether, 3207 such pairs were identified (see Tables 1 and 2 in **Text S1**). This analysis provided a standard for comparing gene functions between *P. aeruginosa* and *P. putida*, as the reciprocal genes were assumed to have the same function in the absence of opposing evidence.

### Initial differences between reconstructions of *P. aeruginosa* and *P. putida*

After identifying reciprocal gene pairs, we sought to identify the number of differences between the two reconstructions, which would reveal the scope of the reconciliation task. This step required identification of common metabolites and reactions for both reconstructions, but as the two metabolic GENRE**s** were built and are maintained by different groups and often incorporate different names for many chemical species and reactions, this step was not completely straightforward and could not be performed entirely automatically. Of the set of 1328 reactions present in both reconstructions, we were unable to find a match in the other reconstruction for 619 reactions; 277 reactions were initially determined to have a functional but not an exact equivalent in the other reconstruction (e.g. two reactions differing only in cofactor usage, but performing *de facto* the same conversion). These numbers are shown in Table 4 in **Text S1**. Of the Gene-Protein Relationships (GPRs: see [18] for an in-depth explanation) of the 432 remaining reactions (all of which were present in both reconstructions), 223 had identical gene associations in both reconstructions (i.e. the reciprocal matches of all genes associated with a given reaction in one reconstruction were associated with the reaction in the other reconstruction and the genes were connected by the same logical expression). These 233 reactions were left alone, since they fulfilled the null hypothesis that no difference exists between the reconstructions for the given reactions. Additionally, a set of 20 reactions were removed from the *P. aeruginosa* reconstruction because they were partially redundant with other reactions in the reconstruction. This left a total of 1074 reactions to be reconciled. These reactions contained differences in reaction stoichiometry, reaction participation, or GPRs between the two reconstructions, and during the reconciliation process each of these differences was investigated and resolved in turn.

### Reconciliation process

Having identified reciprocal gene pairs and outlined the general scope of the reconciliation process, we next developed a system for assessing reactions in the two reconstructions and determining how to best reconcile differences. The process of reconciliation is schematically outlined in **Figure 1**. Each reaction was assessed individually in order to determine if differences between the two reconstructions relating to that reaction were substantiated by experimental, genomic, or other available evidence. First, all genes associated with the reaction were assessed in both reconstructions. This assessment included an evaluation of whether any of these genes were members of a reciprocal pair and, if so, whether the other pair member was also assigned to the reaction. If a discrepancy was found in assignment of the reciprocal pair members to a given reaction, the reason for the discrepancy was identified and this discrepancy was reconciled by either removing one pair member from or adding the other to the GPR of the reaction. In addition to these types of discrepancies, genes not possessing a reciprocal in the other organism were also thoroughly evaluated for their function, in order to avoid creating or retaining apparent differences (e.g., if functionally similar reactions with slightly different stoichiometry were present in iMO1056 and iJP815, but only one had a gene association while the other was added for gap-filling purposes). The decision of how to treat each gene or reciprocal pair was based on gene annotations, functional information from biological databases, and annotations of homologous genes from other organisms. Any literature associated with the reaction during the original reconstruction process was also re-analyzed, and new literature evidence was sought particularly in cases where sequence-based comparisons yielded incomplete or contradictory results. Furthermore, experimentally confirmed physiological phenomena (e.g., from the validation of the original reconstructions) were taken into account in this process. Once the GPR associations had been reconciled for a given reaction, the required changes to the reconstructions were made. These changes could include adding or removing the reaction from one or both reconstructions, or modifying the reaction GPRs. The main factors contributing to decisions about each of the reactions in the reconstructions were recorded in a set of reconciliation notes, which serve as annotations of the reconciliation process for future investigation. The reconciliation notes are provided in Table 5 in **Text S1**.

### Streamlining the reconciliation process

It became apparent in the early implementation of the reconciliation process that certain reactions required different types and layouts of data in order to be reconciled. Therefore, to streamline the reconciliation process, reactions were split into groups, each of which would be reconciled with a slightly different procedure. The reactions were split into main groups as listed in **Table 1**, and the number of reactions and the general process used for each group are indicated. Initially, the groups of reactions to be reconciled were based on patterns of gene associations (e.g., reactions only in one reconstruction, reactions in both reconstructions but with differing GPRs), but it was quickly determined that the reconciliation could be optimized in cases where multiple similar reactions were associated with the same set of genes. Therefore, in addition to information directly relevant to the reconciliation of a reaction (such as reaction stoichiometry and directionality, gene associations and annotated functions), all other reactions in iMO1056 and iJP815 associated with any of the same genes were also considered along with the reaction being reconciled. This consideration of multiple reactions utilizing the same genes assured that the decisions pertaining to one particular gene remain consistent and enabled us, for example, to identify reactions that might have been present in both reconstructions but with slightly different stoichiometries.

**Table 1:** Reconciliation strategy for various groups of reactions.

Category	# Rxns	General Reconciliation Strategy
**Reaction in 1 model only, but genes have reciprocals in other organism**	256	If there's a good BLAST match (>70% identity) and annotations are similar, either include or disclude reactions from both models, depending on whether annotation is sufficient to warrant inclusion. PseudoCAP and KEGG were generally sufficient.
**Reaction in 1 model only, genes don't have reciprocals**	320	List PseudoCAP annotations for the best BLAST matches to the associated genes, their best BLAST matches, and their best BLAST matches. List all reactions in both models that contain any of these genes. Consult KEGG and other sources if the answer is ambiguous.
**Reaction in both models with different numbers of genes in each**	79	List out all best-hit BLAST relationships between genes. Include Pseudocap and/or KEGG annotation information. Assess gene participation gene by gene.
**Association identical in PAO and PPU**	222	No action required.
**Other**	73	Perform literature searches and/or various methods described in other sections.
**Pathway: aldehyde dehydrogenases**	22	Standardize all related reactions to have the same genes in each model, since these enzymes are promiscuous.
**Pathway: amino acid catabolism**	19	Pathway Involves many probable or putative acyl-coa dehydrogenases. List out all genes and annotations involved in this entire group of reactions, and include in both models all reactions containing any of those genes. Crosscheck annotations in KEGG. Be consistent in which genes are kept between reactions.
**Pathway: beta oxidation Pathway**	42	Pathway was not present in initial reconstruction of PAO, so use homology from PPU as guide in reconstruction.
**Pathway: fatty acid synthesis**	26	List all PAO and PPU genes associated with this pathway and their annotations; determine which genes should be associated for the different acyl chain lengths and steps based on PseudoCAP and KEGG. Use KEGG gene associations if PseudoCAP is ambiguous. Standardize reaction stoichiometry.
**Pathway: O-antigen synthesis**	13	New literature reference used to standardize and update the pathway in both models.
**Pathway: lipopolysaccharide**	68	Use BLAST homology and PseudoCAP annotation to determine presence of the various genes in PPU. Use the iMO1056 LPS reactions as basis for the PPU reactions, but make the stoichiometries organism specific based on acyl chain lengths and any other differences seen in literature.
**Pathway: transport**	139	Use BLAST comparisons, TCDB and PseudoCAP to determine reaction stoichiometries and gene participation. Group all reaction-gene families that cross-participate in each other in order to standardize genes associated with multiple transport reactions.
**Possible functional equivalents**	119	Use various data sources to determine which reaction is correct. Standardize reactions if they should be equivalent.

During reconciliation, reactions were categorized into groups based on the types of information that would likely be useful in reconciling them between the two reconstructions. The major categories are listed, as well as the number of reactions (# Rxns) reconciled in each category and the general reconciliation strategy used. When interpreting the ‘# Rxns’ field, note that some reactions that do not ‘fit’ in a given category were nevertheless reconciled in that category, since the reconciliation of many reactions involved cross-referencing and reconciling other reactions with similar gene associations. The ‘Pathway’ categories were created for sets of reactions that did not easily conform to the general methods used for reconciliation, but had unique or specific methods that were found to be more appropriate.

Furthermore, we found that certain pathways would be easier to reconcile if split off into separate groups, regardless of their gene association patterns. This division pertained mainly to linear pathways with few cross-connections to other pathways as well as highly organism-specific processes, such as the pathways for beta-oxidation and lipopolysaccharide (LPS) synthesis. Many of the reactions in the LPS pathway, for example, were reconstructed originally from literature sources rather than from database information, so reconciling this pathway as a group made more sense than breaking the reactions up into groups based on the type of gene associations present.

The panel on the bottom right of **Figure 1** shows data laid out for a reconciliation of the reaction ‘GMP synthase (glutamine hydrolyzing)’, which was initially included in both reconstructions, but with different numbers of genes associated in each model. In iMO1056, there were initially two genes associated (PA1742 and PA3769). In iJP815, only one gene (PP1032) was associated. In the center of the panel, reciprocal gene matches are shown for all genes associated with GMP synthase in either iMO1056 or iJP815. In this case all the assigned genes possess a reciprocal, yet PP1842, the reciprocal of PA1742, was originally not associated with GMP synthase. To determine if the gene annotations corroborated the reciprocal gene pairings, primary gene functions from the PseudoCAP annotation [19] were listed next to each gene. Then, auxiliary information about the genes was collected. In this case, the EC numbers listed in the PseudoCAP annotation were consistent for PA1742 and PP1842 and were the same as that for GMP synthase, contributing to the evidence that these genes both should be associated with GMP synthase. In some cases, various other databases or literature sources were included in this auxiliary section. In this case, the *P. putida* gene PP1842 was added to the reconciled GENRE, since available evidence indicated that this gene, like its reciprocal in *P. aeruginosa*, encodes a protein with GMP synthase activity.

### Assessing the impact of reconciliation on the reconstructions

**Figure 2:**
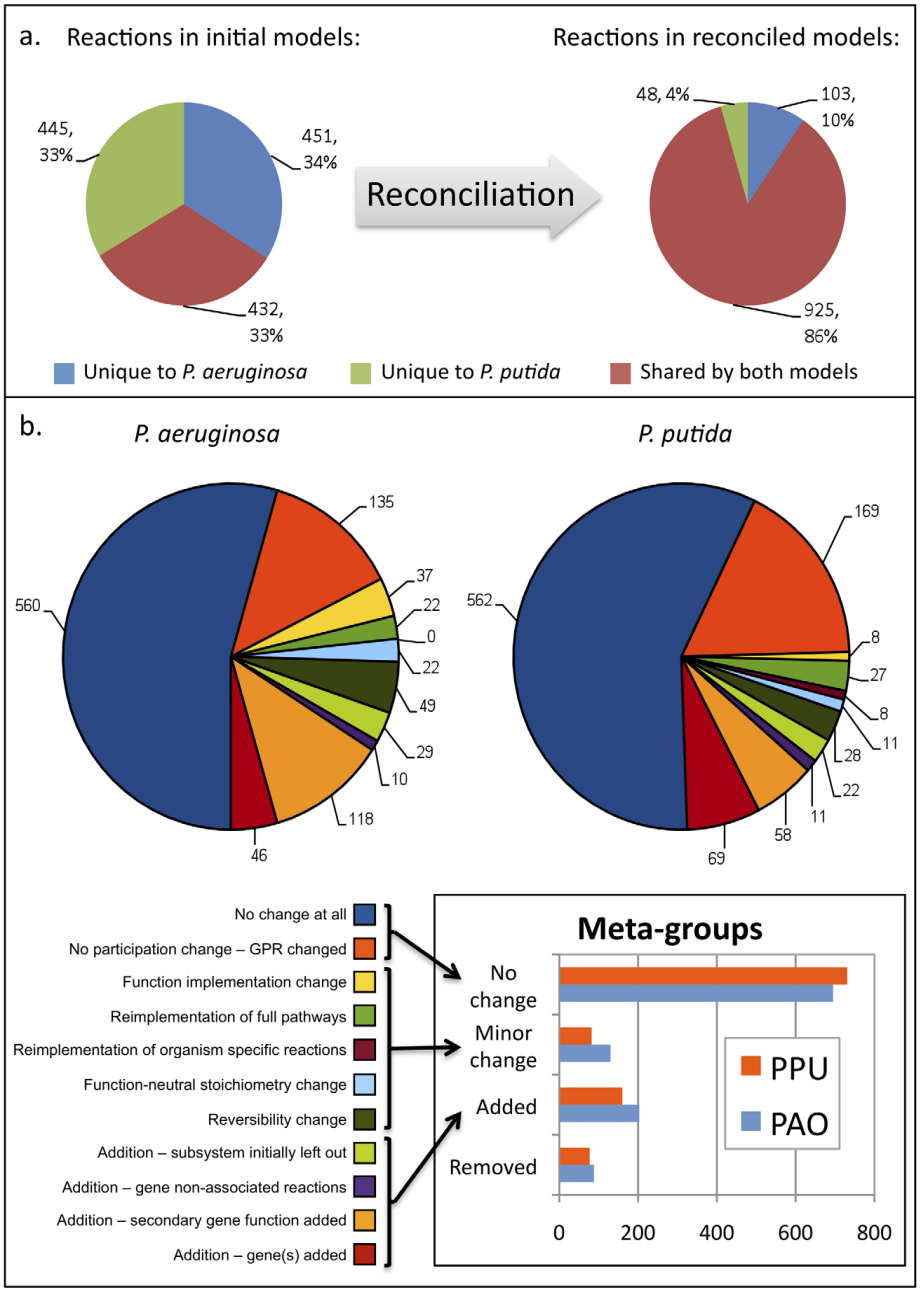
Reconciliation results. This figure highlights statistics of the reaction participation in both the *P. aeruginosa* and *P. putida* reconstructions before and after reconciliation, as well as the reasons for adding or removing reactions to the reconstructions. Panel (**a**) shows the numbers of reactions from both initial reconstructions that are unique to the two organisms versus shared between the reconstructions (left), and the reaction participation following reconciliation (right). Panel (**b**) shows the reasons for changing reactions in both reconstructions. These reasons for changing reactions are then grouped into broader ‘meta-groups’, as shown in the inset plot. In panel (**b**), the colors for the categories and the pie charts are correlated.

The reconciliation process resulted in a large convergence between the *P. aeruginosa* and *P. putida* reconstructions. While the original reconstructions shared 432 reactions, with 451 and 445 reactions unique to iMO1056 and iJP815 respectively, the reconciled final reconstructions (iMO1086 and iJP962 for *P. aeruginosa* and *P. putida*, respectively) share 925 reactions, with only 103 and 48 reactions unique to the each reconstruction (see **Figure 2a**). Although such large changes in the overall statistics suggest that the reconstructions underwent major alterations, a detailed analysis of the types of changes at the reaction level reveals that most of the changes relate to equilibration of similar but non-identical functions, and as a whole do not tremendously alter the biology of the networks. Reactions in the final reconstructions were assigned to classes that best describe their fate during the reconciliation process. These assignments are depicted for both reconstructions in **Figure 2b**. The pie charts, along with the legend (lower left), show the types of changes that all reactions in the final reconstructions went through during reconciliation. These classes can be grouped into four meta-classes, as shown in the inset graph. These classes are: (i) *no change*, describing reactions preserved with unchanged stoichiometry, (but including some reactions that had changes in their GPRs); (ii) *added*, describing reactions added to one of the reconstructions during the reconciliation process, such as in cases where a secondary function of an enzyme had been added to one of the pre-reconciliation models but not to the other, and the reconciliation resulted in the function being included in both; (iii) *removed*, describing reactions removed from the one of the reconstructions during the reconciliation process; and (iv) *minor change*, describing reactions whose general functions were preserved despite modifications to the specific stoichiometries or pathway participation of the reactions involved in the process. This last category includes, for example, reactions in the lipopolysaccharide production pathway, which was preserved in both GENREs but was modified based on a publication that appeared after the original reconstruction processes had been completed [20]. Only reactions present in the final reconstructions are shown in the pie charts in **Figure 2b**, hence the *removed* class is not represented in these charts. The largest of these meta-groups is *no change*, followed by *added*, *minor change*, and then *removed* (see **Figure 2b** **, inset**). A fuller description of the different types of changes made to the models is provided in **Text S2** (see section “Categories of changes made during reconciliation”).

#### Changes in pathway participation

**Figure 3:**
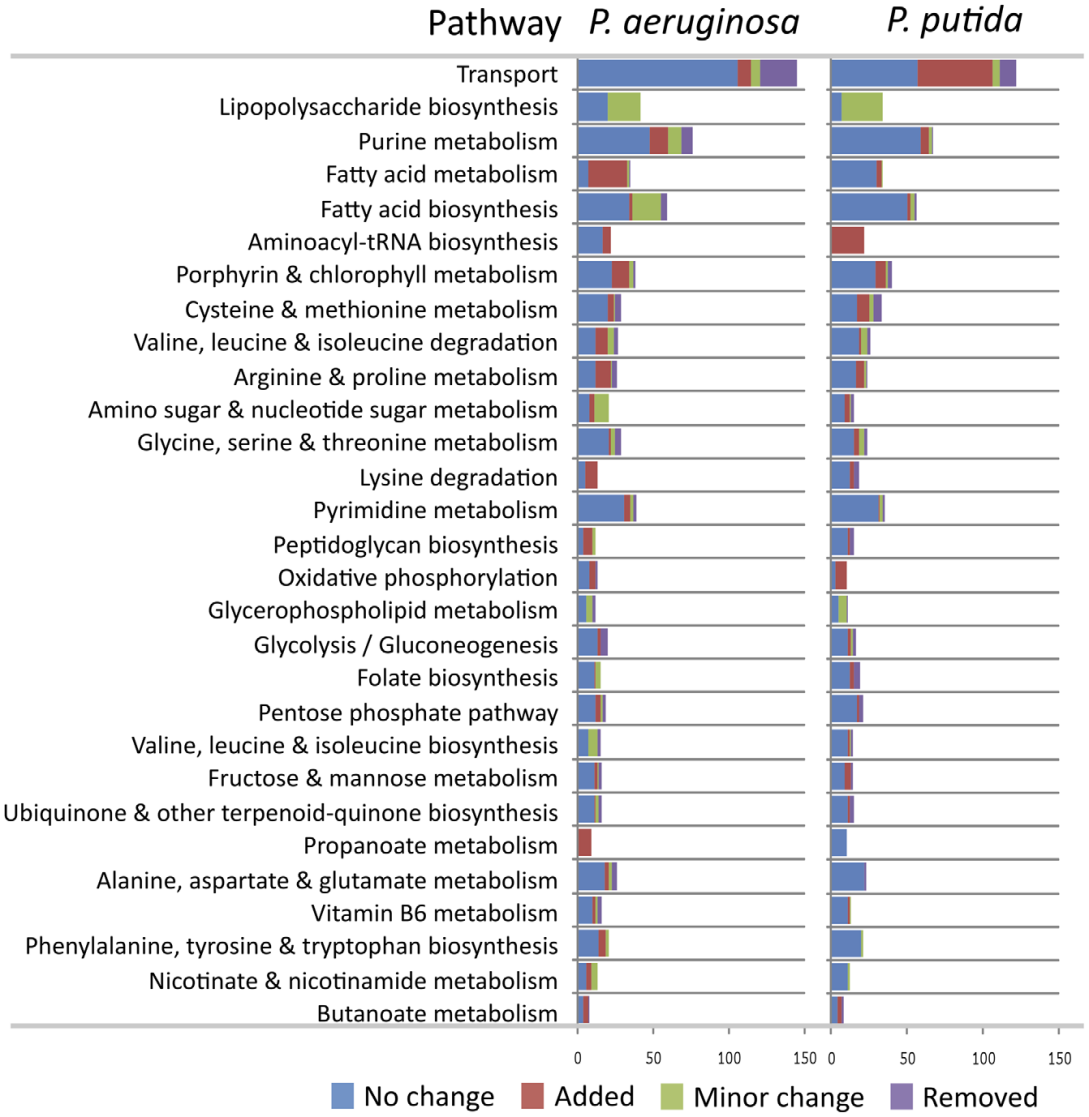
Changes to reconstructions by pathway. The changes made to the *P. putida* and *P. aeruginosa* reconstructions during reconciliation are split into the four ‘meta-groups’ described in **Figure 2**, and listed by pathway. Only pathways with at least eight reactions changed (as a sum of changes in both reconstructions) are listed, and pathways are ranked by the aggregate number of reactions changed during reconciliation.

To gain a sense of where changes occurred in the reconstructions during reconciliation as well as the functional differences present in the reconciled GENREs of *P. aeruginosa* and *P. putida*, we mapped out the KEGG pathways of all reactions in the pre- and post- reconciliation GENREs. **Figure 3** shows the breakdown by pathway of reactions changed in the reconstructions during reconciliation, ranked from most to least altered pathways (pathways with less than 8 cumulative changes are omitted from the plot). This plot reveals which pathways contain the most ambiguous information in available databases for *P. putida* and *P. aeruginosa*, as the pathways with the most changes tend to be enriched in reactions that required subjective decisions during the initial reconstruction processes for iMO1056 and iJP815, and therefore would be subject to more alterations during reconciliation than other more well defined pathways. Transport reactions are the most altered on the list, partially because of the large number of transport enzymes present in *P. aeruginosa* and *P. putida*, but also due to the high degree of ambiguity present in current knowledge of these transporters [12], [21], [22]. Lipopolysaccharide (LPS) synthesis, purine metabolism, and fatty acid metabolism and biosynthesis also contained large numbers of changes, partially due to incorporation of new literature data for less understood pathways (in the case of LPS), as well as ambiguity in the directionalities and functions of certain promiscuous enzymes (in the cases of purine and fatty acid metabolism). This characterization of ambiguity in different pathways was similar to a quantification of ambiguity done in building the human metabolic reconstruction [23], which lends an interesting context to the pathways identified as ‘ambiguous’ in this study. The most striking similarity in ambiguous pathways with the human effort is transport reactions (which are highly ambiguous in both). Transport, it should be noted, is not truly a ‘pathway,’ but rather a functional class of related (but not necessarily connected) reactions, which sets it apart from most of the other reaction groups. Aside from transport, many of the other highly ambiguous processes identified for *P. aeruginosa* and *P. putida* during the reconciliation, including purine metabolism and fatty acid metabolism, fall into the high confidence category in the human network. This is likely a reflection of differences in the knowledge available for human versus bacterial pathways.

#### Metabolic differences between *P. aeruginosa* and *P. putida*

**Figure 4:**
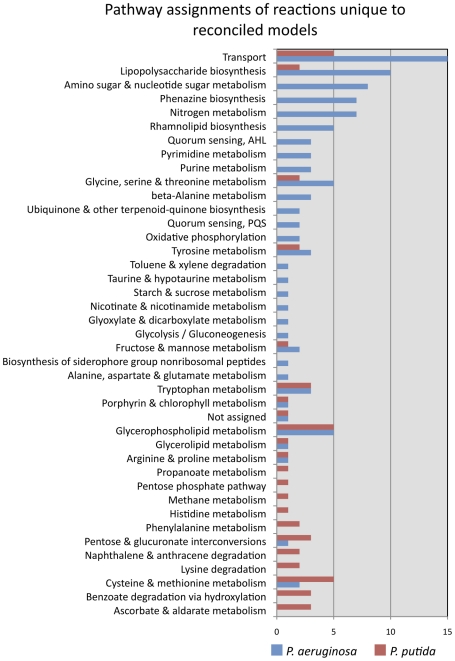
Pathways unique to the reconciled reconstructions. All pathways for which some reactions are present in the reconciled *P. aeruginosa* reconstruction but not the *P. putida* reconstruction, or vice versa, are listed. The size of the bars corresponds to the number of unique reactions in *P. aeruginosa* (red) or *P. putida* (blue).

We were interested in examining the differences in reaction participation between the final (reconciled) *P. aeruginosa* and *P. putida* reconstructions, as these GENREs represent our best understanding of metabolic differences between these organisms. These results can be seen in **Figure 4**, with bars representing the number of unique reactions in the given organism associated with the different KEGG pathways. This analysis confirms several expected results, including the unique assignment to *P. aeruginosa* of reactions for synthesizing quorum sensing molecules, phenazines and rhamnolipids, and for performing denitrification [12], [24], [25], [26], [27]. The capacity to denitrify is partially responsible for the ability of *P. aeruginosa* to thrive anoxically, whereas *P. putida* is a strict aerobe. Unique assignments of several aromatic degradation pathways for *P. putida* are also as expected, including those for degradation of naphthalene, anthracene, phenylalanine, and benzoate [28], [29], [30]. Some pathways, including purine, pyrimidine, and beta-alanine metabolism, revealed unique reactions in *P. aeruginosa* in the reconciled reconstructions. Further, some pathways (e.g., glycerophospholipid metabolism, tryptophan metabolism, cysteine and methionine metabolism, and some others) show a number of unique reactions in both *P. aeruginosa* and in *P. putida*, indicating that these pathways utilize different mechanisms in the two organisms. Overall, more unique reactions appear to belong to *P. aeruginosa* than *P. putida*, and these span a wide variety of pathways involved in many different cellular processes. It is important to note that many hypothetical enzymes exist in both of these organisms, but that *P. putida* has been considerably less well studied than *P. aeruginosa*, which might partially explain why more unique reactions are present in the *P. aeruginosa* reconstruction. However, it is likely that most of the uncharacterized enzymes participate in peripheral metabolism, so the functioning of central metabolism in the two organisms should still be amenable to comparison.

### Experimental validation of reconciled GENREs

After completing the reconciliation process, it was important to re-validate the post-reconciliation GENREs with the same data we had used to validate the original metabolic GENRE**s** of *P. aeruginosa* and *P. putida*. Both original reconstructions had been validated by comparison of *in silico* growth predictions versus BIOLOG substrate utilization data and growth yield data collected from literature, and the *P. aeruginosa* network had been further validated by genome-scale gene essentiality data and *P. putida* against a set of auxotrophic mutations. We compared the reconciled GENREs to the same data to determine how the reconciliation process affected accuracy of the models in predicting *in vitro* phenotypes.

#### BIOLOG validation

**Figure 5:**
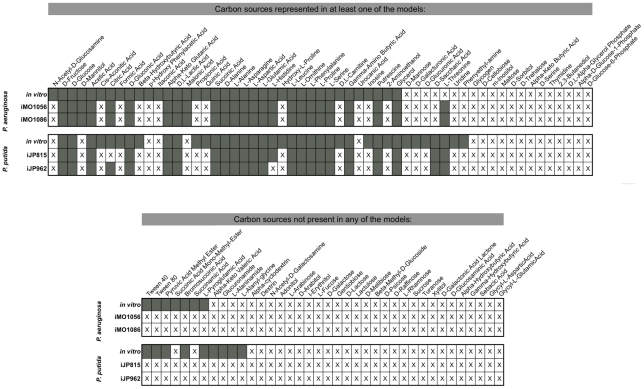
BIOLOG validation of reconciled reconstructions. Substrate utilization results are listed for iMO1086 and iJP962. *In vitro* results from BIOLOG studies of the two organisms are compared to pre- and post- reconciliation network predictions of viability of *P. aeruginosa* and *P. putida* on minimal media with the listed substrate as sole carbon source. The top section of the table includes substrates that are represented in at least one of the metabolic reconstructions (pre- or post- reconciliation GENRE of either organism), and the bottom section includes the remaining substrates assessed in the BIOLOG assay. Substrate utilization is indicated by a box being shaded in, whereas non-utilization is indicated with an X.

BIOLOG phenotyping studies were performed previously for *P. aeruginosa* PAO1 and *P. putida* KT2440 to assess utilization of various carbon sources on otherwise minimal media ([13], [14]). The BIOLOG assay utilizes a tetrazolium dye that is reduced in the presence of strongly respiring cells, leading to a color change that is used as a readout for substrate utilization [31]. In all, the two organisms were tested for utilization of 95 substrates, 49 and 51 of which were accounted for in iMO1056 and iJP815 respectively. The original reconstructions achieved accuracies of 78% and 75% respectively in predicting growth on these various substrates. The accuracies of the reconciled GENREs were exactly unchanged from the initial reconstructions, indicating that changes during the reconciliation did not degrade the ability of the reconstructions to predict *in vitro* phenotypes. Full BIOLOG results and breakdowns of the types of mismatches for both reconstructions are shown in **Figure 5** and in Table 6 in **Text S1**.

#### Growth phenotypes of single-gene mutants

The reconciled reconstruction of *P. putida* was validated by comparison of *in silico* growth predictions versus phenotypes of a set of mutants selected for their inability to grow on acetate as a sole carbon source, as had been done in validation of the initial model [14]. The initial reconstruction (iJP815) correctly predicted the lack of growth in the case of 38 genes, while it failed in 13 cases. Eight of the genes for which mutations had been generated were not included in the iJP815 reconstruction, so the overall accuracy of the reconstruction (including these eight mutants in unaccounted-for genes) was 64%. The reconciled GENRE included one gene more from the set of mutants (PP2001), and made correct predictions in the case of three more genes. This caused an increase in accuracy to 69%. Complete results are given in Table 3 in **Text S1**.

Additionally, we assessed the accuracy of iMO1086 in predicting essentiality of metabolic genes in *P. aeruginosa*, using a list of genes deemed essential in both of two independent genome-scale transposon studies of *P. aeruginosa* as was done in the initial validation of iMO1056 [13], [32], [33]. The reconciliation process only slightly influenced the performance of the reconstruction in the prediction of gene essentiality, as the overall accuracy of the reconciled GENRE decreased by 0.8% from that of the original model (see Table 8 in **Text S1**). It is important to note that there are different sets of genes associated with iMO1056 and iMO1086, with 143 genes unique to iMO1086 and 123 genes unique to iMO1056. An examination of the 933 genes common to both iMO1056 and iMO1086 indicates that essentiality predictions among this set improved marginally (2 genes out of 933; see Table 9 in **Text S1**). This indicates that only slight improvements were made to the GPRs insofar as gene essentiality predictions are concerned, although the improvements might turn out to be more profound if more or different data were available for validation.

The slight improvement that was seen in the essentiality predictions for the 933 genes common to iMO1056 and iMO1086 was the result of an almost exactly balanced deterioration (true positive (TP) → false negative (FN) and true negative (TN) → false positive (FP)) and improvement of calls for approximately 6% of genes (see Table 9 in **Text S1**). The most prevalent conversion (24 genes) was TN→FP, which is in agreement with the overall higher fraction of false positive calls in the reconciled reconstruction. The lack of significant change in accuracy of the predictions of gene essentiality between the pre- and post-reconciliation reconstructions suggests the possibility of an intrinsic limit on accuracy, which would likely differ in different organisms based on the completeness of the genome annotation and other available databases, as well as on the amount of validating experimental data available.

We additionally assessed the impact on essentiality predictions of including cysteine and some nucleotides in the *in silico* LB medium, and found that inclusion of these components improved accuracy of predictions, suggesting that these components were likely present in the *in vitro* LB medium. This analysis is provided in Table 10 in **Text S2**.

#### Central vs. peripheral reconciliation-driven changes in models

The lack of significant changes in the results of model validation between pre- and post- reconciliation reconstructions led us to hypothesize that a high proportion of the changes to the models might have occurred in peripheral pathways that are not directly involved in growth. To test this hypothesis, we optimized the reconciled models of *P. aeruginosa* and *P. putida* for biomass and assessed reaction participation. The minimal sets of reactions essential for any growth, as well as the sets of all reactions that must carry flux to sustain optimal growth, were determined for both organisms under the condition of glucose minimal medium. Reactions that fall into these essential sets represent an unbiased attempt to define ‘central’ reactions, as defined by their involvement in biomass production.

**Figure 6:**
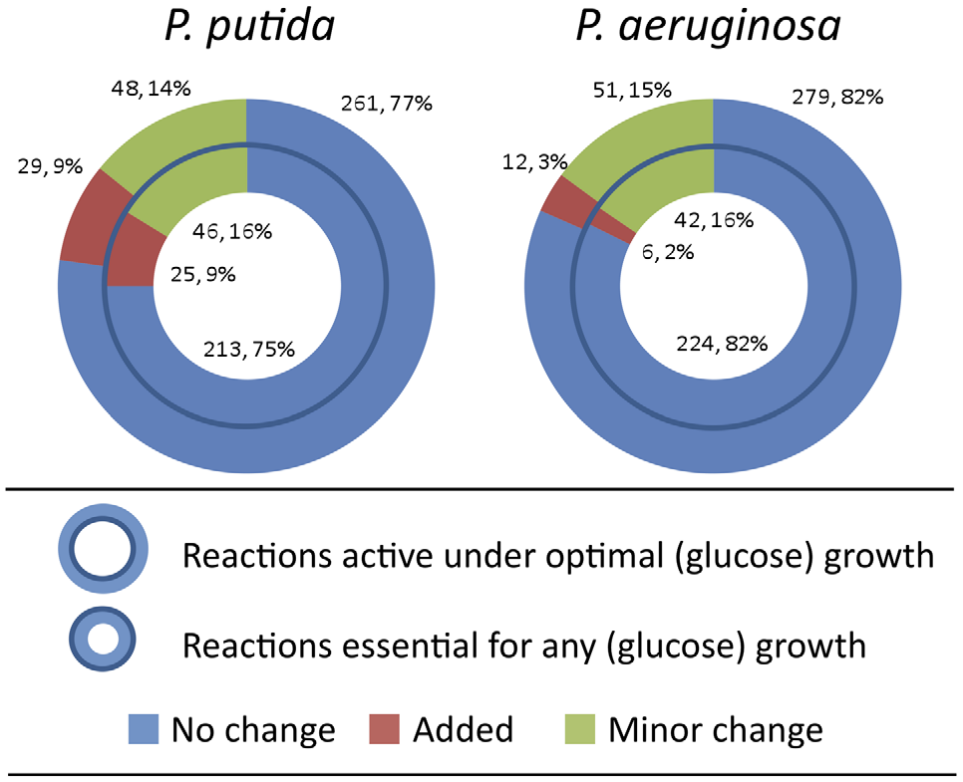
Reconciliation-derived changes in essential reactions. Reactions that are essential in the reconciled models for optimal (outer circles) or any (inner circles) growth were determined in *in silico* glucose medium. The numbers of these reactions belonging to the *no change*, *added*, and *minor change* categories (from the reconciliation) are indicated in the donut charts.

These results, as well as assignments of these reactions to *no change*, *added*, and *minor change* groups from the reconciliation process, are shown in **Figure 6**. The percentages of reactions that were unchanged from the initial models (*no change* category) in these essential reaction sets are approximately equivalent to the percent seen in the model as a whole for *P. putida* (75% and 77% versus 77%; contrast **Figure 6** vs. **Figure 2b**, inset), but in the *P. aeruginosa* model, the percentages of reactions in the *no change* category in the essential reaction sets (82% for both optimally and minimally essential reaction sets; see **Figure 6**) contrast significantly from the 68% of reactions unchanged in the model as a whole. Furthermore, when the *minor change* category is lumped in with the *no change* category, both *P. putida* and *P. aeruginosa* display enrichment of essential reactions in the ‘no change/minor change’ categories by between 8 and 18% over percentages of reactions in these categories from the entire models. This analysis indicates that changes to the reconstructions through the reconciliation process tended to target non-central processes, yet, as will be described below, these changes do affect processes of direct interest for understanding key aspects of the physiology of these bacteria.

#### Predicting yields

To assess how the reconciliation affected the yield predictions of the two metabolic GENREs, we performed flux balance analysis (FBA) simulations for growth of the original and the reconciled reconstructions. In both models, the yield increased slightly from the original models (9.4% and 3.6% for *P. putida* and *P. aeruginosa*, respectively). This increase is due to an increase in P:O ratio and, in the case of *P. putida*, a slight change in the biomass composition from the original model. For a more detailed analysis of yields in the models, see section “Analysis of changes in yields” in **Text S2** and Table 7 in **Text S1**.

### Comparative analysis of *P. aeruginosa* and *P. putida*

#### Analysis of virulence precursor tradeoffs

**Figure 7:**
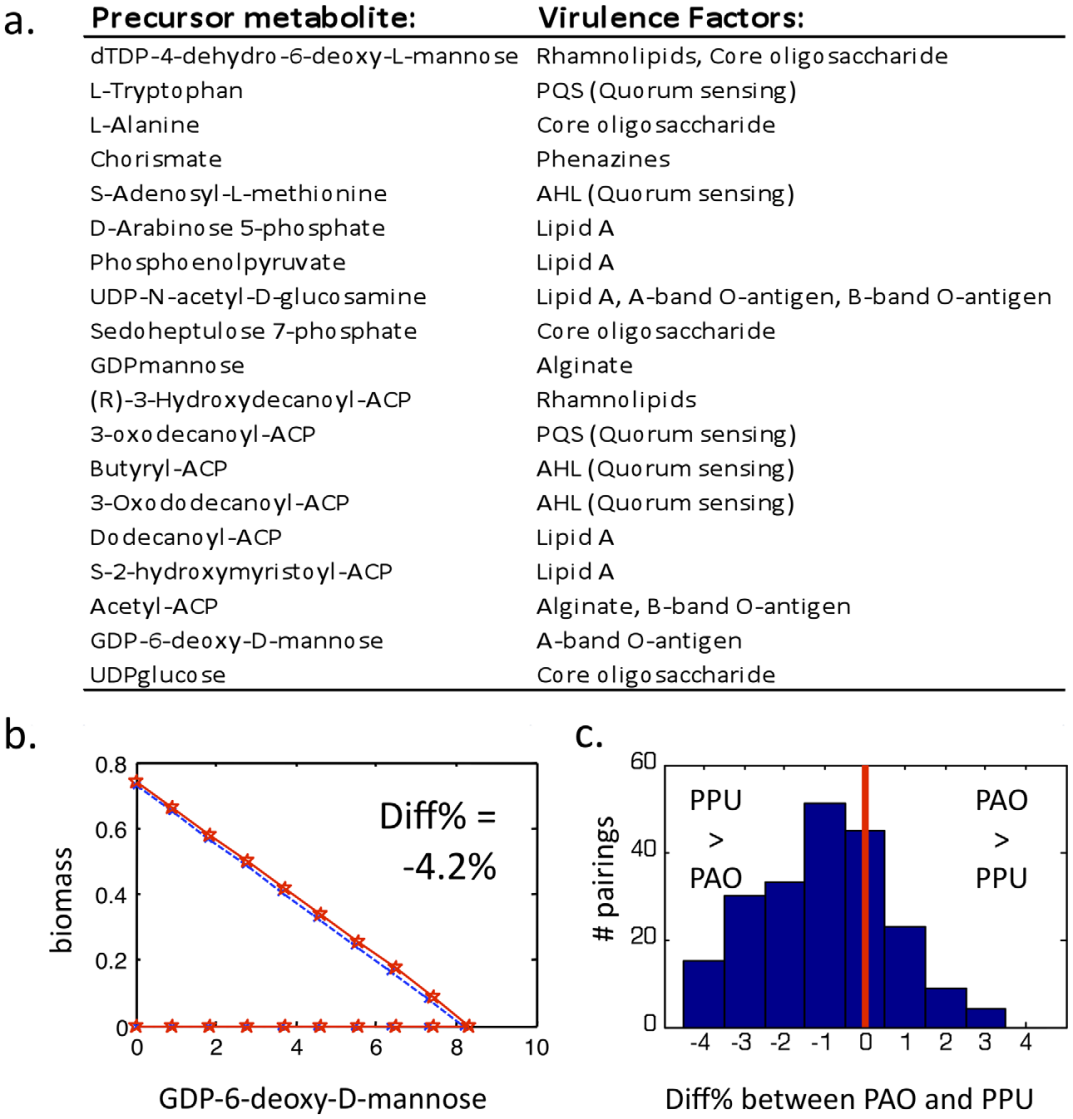
Analysis of tradeoffs in producing virulence factor precursors. Differences were analyzed between *P. aeruginosa* and *P. putida* in their flexibility in tradeoffs between all pairs of 19 virulence factor precursors (plus the biomass reaction). This analysis was done by constructing pareto optimum curves, as described in Materials and Methods. Panel (**a**) lists the 19 precursor metabolites, along with the virulence factors for which they are precursors. Panel (**b**) shows the virulence precursor tradeoff curve that most differs between *P. aeruginosa* and *P. putida*. Units refer to feasible flux values through demand reactions for the given virulence precursor in units of (mmol)·gDW^−1^·h^−1^ given a specific glucose uptake rate of 10 (mmol glc)·gDW^−1^·h^−1^. The percentage by which the areas of these curves differ between iMO1086 and iJP962 is shown in the top right of the plot (with the ‘-’ value indicating a greater area (i.e., greater flexibility) in *P. putida* than in *P. aeruginosa*. Panel (**c**) shows a histogram of all of the reaction pairings, binned based on the percentage difference in flexibility between *P. aeruginosa* and *P. putida*.

The initial reconstruction of *P. aeruginosa* enabled the identification of metabolic precursors for a number of virulence factors (see **Figure 7a**, or Table 1 from [13]). These precursors are all present in both *P. aeruginosa* and *P. putida*, and they represent the last non-committed metabolites in the pathways to produce the virulence factors. We were interested in analyzing differences between *P. aeruginosa* and *P. putida* in how metabolically taxing it is to produce these precursors. One way to measure metabolic capacity is to construct a pareto optimum curve between two possible cellular objectives, which displays the tradeoffs between optimal maximization (or minimization) of the objectives (see [34]). To examine the metabolic demands for producing virulence factor precursors in *P. aeruginosa* versus *P. putida*, we computed pareto curves for all pairwise combinations of 19 virulence precursor demand reactions, as well as the biomass exchange reaction (see Materials and Methods for a more detailed explanation). All simulations were done in *in silico* glucose minimal medium. In comparing pareto curves, a larger area within the pareto curve for one organism versus the other indicates that the metabolism of that organism has more freedom in choosing tradeoffs between the two reactions, and is thus an indicator of greater metabolic flexibility. These 19 virulence factor precursors are listed in **Figure 7a**.

This pareto analysis surprisingly showed only small differences in flexibility of *P. aeruginosa* versus *P. putida* for production of any pair of virulence precursors or biomass. Among the 210 unique pairings of these 20 reactions (19 precursor demand reactions +1 biomass exchange reaction), the biggest difference in area held within the pareto curve for *P. aeruginosa* versus *P. putida* was −4.2%, a negative percentage indicating that the area held within the iMO1086 pareto curve is smaller than that for iJP962 (see Materials and Methods for details). This pairing, which is between biomass production and GDP-6-deoxy-D-mannose demand, is shown in **Figure 7b**. **Figure 7c** shows a histogram of the differential percentages in pareto areas for the 210 pairings, with pairings showing greater flexibility in *P. putida* to the left (negative values) and pairings showing greater flexibility in *P. aeruginosa* to the right (positive values). While the differences between *P. aeruginosa* and *P. putida* in flexibility of virulence precursor production tend to be small (less than 5%), there are significantly more flexible pairings in *P. putida* than in *P. aeruginosa*.

This result suggests that while the difference in ease of production of virulence precursors between *P. aeruginosa* and *P. putida* is small, *P. putida* might have slightly lower metabolic costs to producing these factors. It is notable that many of the virulence factor precursors are fairly central to metabolism, and can be used for a variety of metabolic ends unrelated to virulence. Therefore, the differences in flexibility might relate to selective pressures having to do with non virulence related processes. Still, it is interesting and surprising that *P. putida* shows more flexibility for this functionality.

#### Analysis of pathway flexibility

The results of the pareto analysis of virulence precursors give a glimpse of how *P. aeruginosa* and *P. putida* are able to apportion resources between multiple competing pathways.

To extend this analysis to the genome-scale, we performed a survey of differential flux carrying capacity between *P. aeruginosa* and *P. putida* for all paired combinations of 73 metabolic pathways. To do this, we first constructed pareto optimum curves (as described in the previous section) for 10 randomly chosen pairs of reactions belonging to each pathway pair. All simulations were done on *in silico* glucose minimal medium.

**Figure 8:**
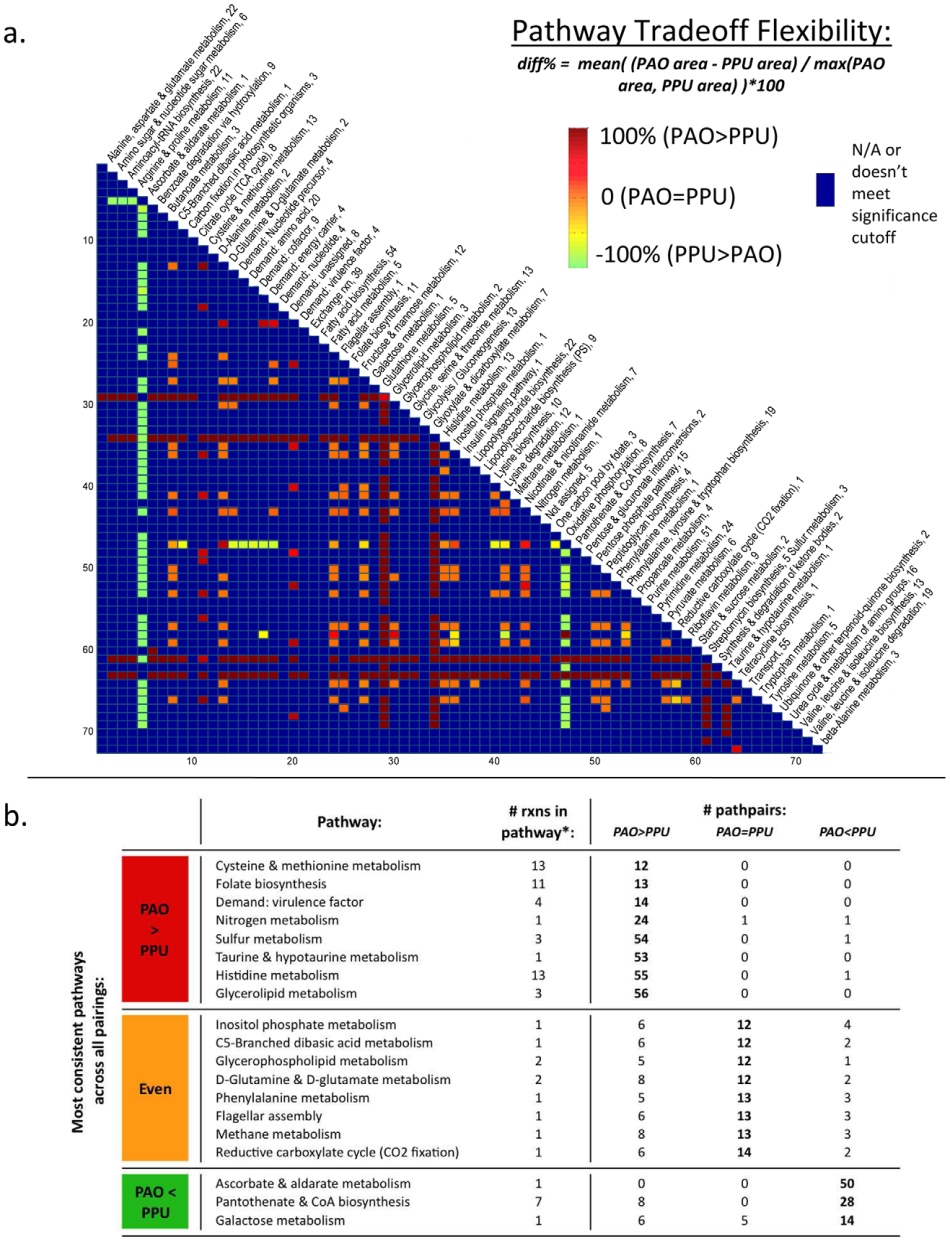
Metabolic flexibility of pathway pairs in PAO and PPU. In panel (**a**) Each pixel represents a pair of pathways in the reconciled models of *P. aeruginosa* (PAO) and *P. putida* (PPU). For each pathway pair, boundary curves for feasible flux tradeoffs were plotted for 20 random pairs of reactions belonging to the two pathways (or 10 pairs for pathways paired with themselves). The area within these feasible flux bounds was calculated in both models for each reaction pair. Then, a statistical test was used to determine whether **PAO** or **PPU** had a significantly larger area in the set of reaction pairs representing the given pathway pair. The degree to which **PAO** or **PPU** showed a larger area for a given pathway pair is marked by pixel color, as shown in the legend. Blue pixels denote pathway pairs that do not meet the significance cutoff, i.e., for which different reaction pairs behaved inconsistently within the pathway pair. A larger area in a given organism can be interpreted as a higher metabolic flexibility with regards to tradeoffs between the reaction/pathway pairs. Panel (**b**) lists features of the pathways with the most flexibility in **PAO** and **PPU**, as derived from the plot in panel (**a**). (*) The number of reactions in each pathway is listed after the name of the pathway in (**a**), and in the ‘# rxns in pathway’ column of (**b**). Only reactions that are both (1) in both models and (2) that can carry flux in at least 1 model are included in this figure.

Next, we utilized a statistical test to determine whether *P. aeruginosa* or *P. putida* displayed a significant degree more flexibility for reaction pairings within each pathway pair (see Materials and Methods for further details). If different reaction pairs in a given pathway pair showed inconsistent results, the pathway pair was deemed not significant. The results of this analysis are shown in **Figure 8a**. This map gives a global view of the difference in flexibility of pathways between *P. aeruginosa* and *P putida* for all pairwise combinations.

Several interesting results are evident from this analysis. In particular, certain pathways are shown to be more flexible in one organism versus the other when compared against virtually any other pathway. Eight pathways in particular display much more flexibility in *P. aeruginosa* versus *P. putida*, while only three pathways display significantly more flexibility in *P. putida* than in *P. aeruginosa*. These pathways are listed in **Figure 8b**. The majority of these pathway pairings differed in area between *P. putida* and *P. aeruginosa* by >50%, with many of them displaying close to 100% more area in one organism versus the other. These results strike an intriguing contrast with the analysis of flexibility in virulence precursors, which displayed at most 5% difference between *P. aeruginosa* and *P. putida*. This result suggests that the metabolic factors that most differentiate *P. aeruginosa* from *P. putida* might not be directly related to virulence factors, but are rather related to other central processes.

Interestingly, three of the pathways indicated to be significantly more flexible in *P. aeruginosa* versus *P. putida* are sulfur-related: ‘sulfur metabolism,’ ‘cysteine and methionine metabolism,’ and ‘taurine and hypotaurine metabolism.’ This result suggests a possible importance of sulfur-related pathways in supporting the different lifestyles of *P. aeruginosa* and *P. putida*. In addition to the sulfur-related pathways, increased flexibility with regards to nitrogen metabolism and virulence factor demand reactions indicate that *P. aeruginosa* is more flexible in areas that are directly related to the habitat of the CF lung and to known virulence determinants. *P. aeruginosa* is capable of performing denitrification, which is often important in the microaerobic conditions of the CF lung [35], [36]. *P. putida*, on the other hand, cannot perform denitrification, nor can it survive anaerobically [12]. The increased flexibility of *P. aeruginosa* nitrogen metabolism over that of *P. putida* is therefore consistent with the known importance of denitrification for virulence.

Increased flexibility in flux through virulence factor demand reactions represents a direct metabolic advantage of *P. aeruginosa* over *P. putida* in factors essential to virulence as well. A puzzling aspect of this result, however, is the contrast between the increased flexibility of *P. aeruginosa* versus *P. putida* in virulence factor production reactions (i.e., demand reactions), but the more similar flexibility between the two organisms in production of virulence factor precursors, as shown in the previous section (areas of pareto curves for the precursors differ by less than 5%, while certain pairings of pathways against virulence factor demand reactions have as high as 100% difference, as shown in **Figure 8a**). The three virulence demand reactions common to iMO1086 and iJP962 (and therefore included in this analysis) are demand for acetylated alginate and for the two homoserine lactones (HSLs) that form the basis of quorum sensing in *P. aeruginosa* (3-oxododecanoyl-HSL and butyryl-HSL). Tradeoffs between production of precursors for these same virulence factors showed minor and mixed differences in flexibility between *P. aeruginosa* and *P. putida*, as shown with the comparison of differential percentages in **Figure 8** versus those in **Figure 7**. This result emphasizes the importance of systems analysis in understanding whole-cell metabolic processes, as the intuitive assumption that demand for virulence factors is merely the sum of demands for individual precursors notably fails in differential analysis of *P. aeruginosa* and *P. putida*.

Several other processes that display more flexibility in iMO1086 versus iJP962 are more difficult to link to a specific virulence trait, but support the general observation that *P. aeruginosa* appears to display greater metabolic flexibility than *P. putida* in a variety of pathways. Greater flexibility was seen in iMO1086 versus iJP962 in 267 pathway pairings, as opposed to 98 pathway pairings in which iJP962 displayed greater flexibility than iMO1086. The most notable exception to the observation of greater flexibility in *P. aeruginosa* than in *P. putida* is the ascorbate and aldarate metabolism pathway, which contributes 50 of the 98 pathway pairings that are significantly more flexible in *P. putida* than in *P. aeruginosa*. It is unclear what biological role a greater flexibility in this pathway would play, particularly as related to virulence phenotypes.

It is likely that many more interesting results are embedded within the pathways deemed non-significant through this analysis, as the cause for non-significance in many cases is the grouping of reactions into a single KEGG pathway when these reactions do not necessarily act in a biologically concerted manner. An unbiased modularization of metabolic pathways might therefore reveal more differences between *P. aeruginosa* and *P. putida* metabolism, but for the purpose of clarity we have restricted our analysis to accepted KEGG pathways.

## Discussion

Comparison of genome-scale metabolic reconstructions (GENREs) and the subsequent comparison of the metabolic phenotypes available to different organisms is a novel method that promises to yield fresh insights into the functioning of metabolism as a whole. Despite the large number of manually curated genome-scale metabolic reconstructions built to date (more than 50), a conspicuous gap exists in the literature for meaningful comparisons between two or more reconstructions of related species [2]. Some comparisons have been done in the past, but they either focus on small subsets of metabolism, utilize automatically generated and thus less well-curated reconstructions, or highlight summary statistics (e.g., numbers of nodes and edges, average path lengths) rather than leveraging the full toolset available for analysis of constraint-based models, which includes flux balance analysis, extreme pathway analysis, and other related methods [2]. Part of the reason why such genome-scale comparison efforts have not been undertaken or have not succeeded yet lies, in our opinion, in the lack of standards for both the software solutions used for the reconstruction process and the reconstruction process itself. This lack of standards causes an inconsistency in the relationships between different metabolic GENREs and the data used to reconstruct them, thus obscuring the valuable information that could be extracted from the comparison of multiple GENREs.

### A workflow for network reconciliation

In order to overcome this hurdle and to enable a high-quality constraint-based comparative analysis of two organisms at genome scale, we developed a novel process termed metabolic network reconciliation. Reconciliation assesses reactions present in two genome-scale reconstructions, and determines whether or not changes should be made to the reconstructions in order to uphold the null hypothesis that no difference exists between the reconstructions. Differences between the two reconstructions for a given reaction are only upheld if sufficient (preferably experimental) evidence for the differences is present in literature, genome annotations, or online databases such as KEGG and Expasy. In this way, the reconciliation allows for verification of two reconstructions against each other and leads to removal of non-verifiable differences between the metabolic reconstructions, while still staying within the bounds of available knowledge about the organisms. We performed the reconciliation between manually-curated metabolic GENREs of *P. aeruginosa* and *P. putida*. These bacteria are both important for biotechnological applications, in addition to the notorious role of *P. aeruginosa* as an opportunistic pathogen. The biotechnological usefulness of these organisms is based on their extremely varied and expansive metabolic capabilities [14], [27], [37], [38].

The reconciliation process resulted in a significant alignment of the *P. aeruginosa* and *P. putida* reconstructions. While in the original reconstructions only around 50% of the reactions were in common, the reconciled GENREs shared around 90% of their reactions. Furthermore, although a significant portion of the reactions in the reconstructions were altered, added, or removed during the reconciliation process, it is clear from **Figure 2b** that most of these changes reflected either an aligning of reciprocal functions (as represented by the *minor change* meta-class) or an addition of pathways that had initially only been reconstructed in one model despite evidence for inclusion in both (as represented by most of the subcategories in the *added* meta-class). Few of the changes altered the fundamental biology of the networks, but these changes were critical in aligning similar functions in the reconstructions so that they could be meaningfully compared. If a comparison between *P. aeruginosa* and *P. putida* had been attempted without first reconciling the metabolic reconstructions, any biological variance between the bacteria would likely have been obscured by differences in the reconstructions derived from the respective reconstruction processes rather than from true biological differences between the organisms.

The similar performance of the pre- versus post- reconciliation reconstructions as compared to validating data suggests that the reconciliation did not alter the overall biology of the reconstructions. However, as all analyses performed in the revalidation process relied on FBA with a growth objective, these validating analyses inevitably focus on the portions of the metabolic networks related to production of biomass and utilization of particular substrates. The fact that the changes in network validation were minor suggests that few functional changes occurred in the central portions of the reconstructions, but more functional changes might have occurred in more peripheral portions of metabolism. This hypothesis is consistent with our analysis of reconciliation-derived changes in the essential portions of the reconciled GENREs, as shown in **Figure 6**. Since the comparison of the metabolic networks will likely focus on differences in peripheral functions (where *P. aeruginosa* and *P. putida* most strongly diverge phenotypically), the network reconciliation might have played a larger role in removing non-verifiable functional differences between *P. aeruginosa* and *P. putida* than is suggested by the phenotypic stability of the pre- and post- reconciliation reconstructions with regards to validating data.

### Reconstruction spaces

In addition to contributing to our understanding of *Pseudomonas* metabolism, the reconciliation process offers a unique opportunity to critique and examine the metabolic reconstruction process itself. Some of the changes in the metabolic GENRE**s** through the reconciliation process rectify inevitable mistakes in the original reconstructions, yet the reconciled reconstructions for *P. aeruginosa* and *P. putida* were built with essentially the same data as the original reconstructions, with the additional constraint that decisions on inclusion of genes must be consistent between the two networks. Therefore, in many cases, the changes are more reflective of ambiguity as to what evidence should be considered sufficient for genes to be included in a reconstruction, or what exact form the reactions they catalyze should take.

When a reconstruction is built, the weights given to different data sources and the determination of cutoffs for inclusion of genes in the GENRE inevitably involve a degree of subjectivity, since a large amount of available gene annotation data is too vague to enable definitive determinations of gene function. The lack of changes in the validation outcome indicates that the original and reconciled reconstructions reflect phenotypes of the respective organism equally well. Therefore, there seems to be a degree of ambiguity in the genetic makeup of a reconstruction that is not fully represented in the phenotypes. This ambiguity can be described in the terms of a space of comparably accurate reconstructions of a particular organism, where the pre- and post- reconciliation reconstructions represent points in the space. The size of this ‘reconstruction space’ for a given organism is related to the degree of vagueness in functional assignments of genes, a metric for uncertainty in the current state of knowledge of the organism.

The plots in **Figure 3** therefore give an estimate of the pathways contributing most strongly to the breadth of this ‘reconstruction space,’ as pathways changing the most during reconciliation are often also the ones whose functions are the most ambiguous based on the current knowledge in databases/literature. Examination of the portions of the reconstructions that changed most during reconciliation may therefore provide a roadmap for future improvement of these metabolic reconstructions. As more data are amassed about the metabolism of an organism and integrated into a reconstruction, the space of possible reconstructions will shrink and become a more accurate representation of the actual metabolism of the organism. This is the aim of the iterative model building and validating process.

It would be also interesting to reconcile the *P. putida* reconstruction with the other independently performed GENREs of this organism [38], [39]. This analysis could allow for further assessment of the noise related to the reconstruction process and would surely contribute to the creation of an even more exact model of the bacterium, as well as further defining the shape of the reconstruction space for this organism.

### Standardization of cutoffs and methods

An issue that contributes greatly to the difficulty of building and comparing metabolic reconstructions is the lack of standardization of the methods used for the reconstruction process. This difficulty, which is the basis for the work presented here, has also catalyzed several efforts towards developing common standards for metabolic reconstructions. One approach is based on reconstruction ‘jamborees’, in which communities of biologists gather over several days for an intensive session of effort to improve and standardize a given metabolic GENRE. Jamborees have been held thus far for *Saccharomyces cerevisiae* [40], *Homo Sapiens*, and *Salmonella*, for each of which there are multiple independent reconstructions available (with significant variance in size and metabolic capability), and these meetings have resulted in progress in the standardization and improvement of metabolic reconstructions [4]. Another attempt at standardization is represented by the SEED project (www.theseed.org) [41], in which databases of various metabolic subsystems are maintained by dedicated scientists, thus assuring the coherence between reconstructions of each subsystem in the metabolic GENREs of different organisms. A further effort is that of Microme (www.microme.eu), a large project that aims to extend the scope of microbial genome annotation from functional assignment at gene level to the systematic generation of pathways assemblies and genome-scale metabolic reconstructions, with an initial focus on bacteria. Even with the efforts from jamborees, SEED, and Microme, ambiguity in the evidence used to build metabolic GENRE**s** is unavoidable. Therefore, some type of reconciliation will likely be necessary prior to any multi-reconstruction comparison.

Even in an ideal case where the knowledge about an organism is complete, there still remains some ambiguous decisions in the reconstruction process resulting from the core approximations of constraint-based modeling, which confine the fundamentally analog nature of biology to digital categorizations (e.g. a continuum of enzyme thermodynamics is categorized into ‘reversible’ and ‘non-reversible,’ a continuum of substrate affinities is converted into ‘yes’ or ‘no’ decisions on which metabolites can be acted on by an enzyme, etc.). With regards to this characteristic, metabolic reconstructions are akin to other types of biological models.

### Multi-species comparisons and automation

The reconciliation process reveals challenges that might arise if a high quality comparison is to be performed between more than two species. Reconciling more than two models might prove difficult, since the addition of more models beyond two adds more degrees of freedom to the task. The issues faced in these cases will be similar to those faced in standardization efforts such as SEED, in which some organism specificity is forfeited in favor of model standardization. Our reconciliation process can inform these efforts, and the reconciled models can serve as a gold standard against which these automated reconstruction platforms can be compared.

Although we performed the reconciliation of *P. aeruginosa* and *P. putida* manually, much of the process could be automated for future efforts. The workflow developed for performing model reconciliation (see **Figure 1** and **Text S2**, Figure 1), coupled with knowledge of the specific information types that are important for making decisions about each given reaction (see **Table 1**) and the specific database structures for performing the reconciliation (see Figures 2–4 in **Text S2**) will help make much of the process automatable in the future.

The reconciliation we performed was for two species that are closely related. However, the process we developed should be equally applicable to more distant species if a comparison is to be attempted. Model reconciliation enables true differences in two metabolic networks to be identified above the noise. This identification enables a more confident comparison of differences, which is crucial whether or not the species are closely related. In comparing species that are more distantly related, more pathways might fall into the ‘organism specific’ categories, and thus need to be reconciled as contiguous pathways as opposed to as collections of reactions (see **Table 1**). This category will include the pathways that most diverge between the species being compared, where whole blocks of reactions might be different between one organism and the other. However, the reconciliation process will be similar in structure, and the insights gained in this study should serve as a guide for such attempts. Rigorous genome-scale, multiple-species comparisons are crucial for the elucidation of the evolution of cellular networks and of the underlying genotype-phenotype relationships. The availability of pathway assemblies and metabolic models for a large variety of microbial species would pave the way for new types of comparative and phylogenomic studies. Furthermore, process-based comparisons would enable the identification of system-wide properties that cannot be detected by a simple comparison of gene annotations and will allow the connection of genotype with phenotypic properties at a phylogenetic level. These phenotypes might include habitat specificity, ecological niche information, and the structure of metabolic systems properties that might be (re)engineered.

### Comparative analysis of *P. aeruginosa* and *P. putida*

The results of the comparative analysis lent significant insight into differences between *P. aeruginosa* and *P. putida* that might relate to the marked difference in virulence. Virulence of *P. aeruginosa* is derived from many sources, including its ability to produce a host of specific virulence factors and toxins [42] and its possession of deadly pathogenicity islands [43], [44]. In contrast, although there are rare cases of *P. putida* infections in humans [45], *P. putida* is typically not virulent. It is of note that *P. putida* does not grow well at 37°C (human body temperature), while *P. aeruginosa* thrives at this temperature [46]. *P. putida* also lacks many of the factors necessary for establishing a human infection, including many of the virulence factors possessed by *P. aeruginosa*. Metabolic factors might play a role in forming some of these differential phenotypes.

One unexpected difference identified through our metabolic comparison that might contribute to the virulence of *P. aeruginosa* versus *P. putida* is the increased flexibility of *P. aeruginosa* in sulfur related pathways (see **Figure 8**). Uptake and conversion of sulfate esters or carbon bonded sulfate derivatives into inorganic sulfate—the form of sulfur used by plants for production of cysteine and other essential sulfur containing compounds—is an important process performed in the rhizosphere by bacteria, including *Pseudomonads* [47]. Sulfur metabolism is an important process for both *P. aeruginosa* and *P. putida*, based on its important role in this common habitat. However, it has also been shown that a significantly different but also sulfur rich environment can be found in the CF lung, where mucin (the family of glycosylated proteins forming the basis of mucus) forms a major source of nutrition for *P. aeruginosa*. Several findings in literature support the importance of sulfated mucin in defining the *in vivo* environment in the CF lung, including the enhancement of mucin sulfation in CF patients versus in the normal lung, the presence of multiple highly regulated sulfur uptake mechanisms in *P. aeruginosa* that respond differently to the presence of inorganic sulfate versus the presence of more complex organosulfur compounds, and most notably, the demonstrated ability of *P. aeruginosa* CF isolates to utilize mucin as a sole sulfur source [48], [49]. While the specific reactions that extract sulfur from complex organic sources are not represented in the *P. aeruginosa* reconstruction, this set of pathways is possibly of great import for *P. aeruginosa* virulence, and is a possible important area of study for the future.

Taken as a whole, the results of this study uphold the observation that the virulence of *P. aeruginosa* is highly multifactorial [50]. Flexibility of certain pathways is larger in *P. aeruginosa* than in *P. putida* as shown through the pairwise pathway flexibility study, but this result does not extend to virulence precursors. More puzzling is the observation that *P. aeruginosa* displays higher pathway flexibility in production of certain virulence factors than *P. putida*, even though the flexibility of production of the virulence precursors remains similar between the two (compare **Figure 7b–c** versus the ‘Demand: virulence factor’ row/column in **Figure 8a**). This observation bolsters the ‘multifactorial’ hypothesis, even within a metabolically-focused analysis.

With the reconciled models presented in this study, we have opened an opportunity to gain a deeper understanding of both the difference in virulence of these organisms, as well as the different metabolic features that they possess with relation to metabolic engineering applications. Reconciled genome-scale models of these two organisms will therefore show many uses beyond the analyses of virulence precursor and pathway flexibility presented here.

## Materials and Methods

### Identification of pairs of reciprocal genes – genome-wide BLAST search

The identification of pairs of reciprocal genes from *P. aeruginosa* and *P. putida* was performed with WU BLAST software version 2.0 (© Gish, W., 1996–2003, http://blast.wustl.edu). Nucleotide sequences for all *P. aeruginosa* and *P. putida* genes were downloaded from the *Pseudomonas* Genome Database V2 (http://v2.pseudomonas.com), and the identification was performed as described in the results section.

### Gene essentiality revalidation for iMO1086

The gene essentiality validation followed the same procedure as in the original validation [13]. Subsequently, the influence of the *in silico* rich medium composition on the outcome essentiality analysis was evaluated. The original *in silico* rich medium did not contain L-cysteine but rather it contained L-cystine, a dimeric amino acid composed of two cysteine residues linked by a disulfide bond. Since it is possible that this compound can be broken down into its L-cysteine form and utilized, the influence of the inclusion of L-cysteine into the medium was evaluated. Furthermore, the original *in silico* medium did not contain any sources of purines or pyrimidines. The decision to exclude these compounds from the medium was based on the listed chemical composition of LB medium, as listed in the supplementary materials from [51]. However, evidence from [52] indicates presence of these molecules in LB medium, so *in silico* growth was also assessed in LB medium containing purines/pyrimidines that could be taken up by the reconciled reconstructions (cytosine, 5′-deoxyadenosine, and uracil).

### Re-validation of the reconstructions against BIOLOG phenotyping assays

The revalidation was performed as in the original validation [13], [14]. Briefly, FBA simulations were performed for both reconstructions in *in silico* minimal media, with various single carbon sources allowed into the models. If biomass could be produced with a nonzero flux on a given carbon source (above a threshold that allows for precision/rounding error), then that carbon source was considered to enable growth *in silico*.

### Re-validation of iJP962 against a set of auxotrophic mutants

The revalidation was performed as in the original publication [14]. Briefly, FBA was performed with acetate as a sole *in silico* carbon source, maximizing biomass production. This simulation was done with each of the set of genes causing acetate-auxotrophy knocked out *in silico* to determine biomass yield. If no biomass was produced, the mutation was considered lethal on acetate medium. These *in silico* results were compared against *in vitro* results collected previously by our group [14].

### Yield comparisons between the original and reconciled reconstructions

The yields were computed by performing FBA [53] with the biomass production as the objective, while setting the NGAM parameter (specifically, the minimal flux of the respective ATP dissipation reaction that accounts for NGAM) to zero and the GAM parameter (by setting the appropriate stoichiometry of the reactions accounting for it) to the value used in the original reconstruction. Subsequently the obtained biomass production rates were divided by the upper bound on the glucose uptake rate (10 mmol·g_DW_^−1^·h^−1^) in order to obtain yields (in units of g_DW_·(mmol glucose) ^−1^). For more details, see section “Analysis of changes in yields” in **Text S2**.

### Pareto optimum curves

Pareto optimum curves denote the outline of the solution space of a metabolic network along the plane defined by two reaction fluxes. To construct a pareto optimum curve for two reactions, the flux through one reaction was fixed at a series of different levels, and the flux through the other reaction was both maximized and minimized at each level. These upper and lower bounds corresponding to each flux value of the first reaction gave shape to two pareto curves, which together outline the edges of the solution space along the plane defined by the two reactions. The area held within a pareto curve was calculated as a metric of metabolic flexibility in trading off resources between the two cellular objectives represented by the two reactions plotted.

### Pathway flexibility analysis

An analysis was performed to examine the differences in flexibility for all unique pairings of pathways present in both iJP962 and iMO1086. First, reactions in both models were assigned to pathways based on KEGG assignments with some manual assistance (e.g., for demand reactions). ‘Demand’ pathways encompass reactions enabling drainage of certain interesting cellular metabolites from the metabolic network, and these reactions were only enabled for the purpose of the specific simulations they participated in (they are not generally considered parts of the models). Reactions were sampled from the set of reactions common to both models and able to carry flux in at least one of the models (there were 656 such reactions). The numbers of reactions meeting these criteria for each pathway are listed after the pathway names in **Figure 8**.

To generate the plot shown in **Figure 8**, pareto curves were generated for both iMO1086 and iJP962 for twenty randomly chosen reaction pairs belonging to each pathway pair (or ten reaction pairs in the cases where pathways were paired with themselves), with glucose allowed as the sole carbon source. Areas were computed for each of the sampled pareto curves. Next, a multiple-testing significance test was used to determine whether a significant trend existed among the results for a given pathway pair. Namely, areas computed for the 20 pairs of reactions for a given pathway pair (10 reaction pairs computed for *P. aeruginosa* and (the same) 10 computed for *P. putida*) were randomly assigned to two groups in 1000 permutations, and cases where the true groupings of pareto areas into *P. aeruginosa* and *P. putida* groups showed a mean difference of at least 3 standard deviations from the average of random permutations of groupings of the 40 reactions were considered ‘significant.’

### Technical computing methods

Simulations were done on a PC using the Cobra Toolbox [54] with either Matlab (MathworksInc., Natick, MA, USA) or Octave (http://www.gnu.org/software/octave/). Linear optimizations were performed utilizing either the free GLPK (http://www.gnu.org/software/glpk/) or CPLEX (IBM, Armonk, NY, USA) linear programming solvers, except for the pathway flexibility study, which was performed using Gurobi (http://www.gurobi.com/). The reconstructions were stored and maintained in Excel and MATLAB and the ToBiN platform [14]. The SBML files were generated using an appropriate Perl script. Analyses were done in Matlab, OpenOffice Calc, and MS Excel.

## Supporting Information

Text S1 Supplementary tables.
(10.03 MB XLS)

Text S2 Supplementary information file.
(0.24 MB DOC)
