# Supplementary material for: Reconciliation of Genome-Scale Metabolic Reconstructions for Comparative Systems Analysis
Source: PLoS Comput Biol. 2011 Mar 31;7(3):e1001116. doi: 10.1371/journal.pcbi.1001116 (PMC3068926; doi:10.1371/journal.pcbi.1001116)
Supplement: Text S2 — Supplementary information file. (0.24 MB DOC) [file pcbi.1001116.s002.doc]

**COMPARATIVE SYSTEMS ANALYSIS OF GENOME-SCALE METABOLIC RECONSTRUCTIONS, Supplementary materials:**

**Contents**

[I. Technical implementation of reconciliation 1](#__RefHeading___Toc279412716)

[II. Categories of Changes made during reconciliation 9](#__RefHeading___Toc279412717)

[III. Re-examination of the in silico LB medium 12](#__RefHeading___Toc279412718)

[IV. Analysis of changes in yields 12](#__RefHeading___Toc279412719)

[V. References 14](#__RefHeading___Toc279412720)

# I. Technical implementation of reconciliation

In designing the reconciliation process, we were confronted with an initial decision about how to proceed through differences between the two models. Two basic approaches were possible, and each of these approaches was initially tried with varied results. These approaches were to reconcile differences in the models gene by gene (Figure 1a in Text S2), versus performing the reconciliation reaction by reaction (Figure 1b in Text S2). In each case, the homology scores from our genome-wide BLAST study would be used to link genes in PAO with genes in PPU and vice versa, but the approaches differed in how we proceeded through the reconciliation task. Ultimately, through trial and error, it was determined that the ‘reconciliation by reaction’ approach was favorable, since ultimately it was reaction participation that dictated the function of the models, not gene participation, and because technically and theoretically it turned out to be simpler. However, information from the gene-based approach (e.g. listings of reactions that each gene participated in in the models) was also deemed valuable to the reconciliation process, since often this information could aid in standardizing actions on similar reactions and decrease redundancy when multiple reactions were present in the models for the same set of genes. Therefore, we finally opted for a third ‘mixed’ approach, in which we reconciled the models reaction by reaction, but in reconciling a given reaction, also took into account any other reactions that had similar gene associations. Figure 1c in Text S2 shows this process for a hypothetical reaction ‘rxn1’, which is considered alongside ‘rxn2’ and ‘rxn3’ during reconciliation since they both share gene associations.

|  |
| --- |
| **Figure 1**: Methods for comparing models: by gene vs. by reaction. **(a)** Gene method. **(b)** Reaction method. **(c)** Reaction method, taking into account related reactions. This third, combined method was employed during the reconciliation. |

Implementation of the reconciliation process was done in Microsoft Office Excel, or in OpenOffice. In order to gather the necessary data for each reconciliation decision, several preliminary tables were created as separate worksheets in a master excel file. Representative subsections of the main tables are shown in Figure 2 in Text S2 for the PAO model, and equivalent tables were also generated for PPU. This task could also have been accomplished using databases, and in fact took the form of a database system in terms of how we used the various tables.

|  |
| --- |
| **Figure 2: Worksheets useful for analysis. (a)** iMO1056, lists all reactions in the initial PAO model, indexed by tobin #. Includes reaction stoichiometry, gene associations, etc. (b) iMO1056_locuslisted, lists all genes in the initial PAO model and the reactions in which they participate. **(c)** paovsppu, lists all PAO genes and their BLAST associations with respect to PPU. **(d)** iMO1056_metabolites, lists metabolites in iMO1056, along with their chemical formulas and other information. **(e)** PAOannotation, lists all of the fields of the PseudoCAP annotation for PAO. Note, only the PAO version of each worksheet is described, but similar worksheets for PPU were also employed. |

The ‘primary key’ that we used to refer to reactions in the system was a TOBIN number, so named after the computational platform developed in and utilized by Dr. dos Santos’ laboratory for constraint based modeling applications. Any unique code for each reaction stoichiometry would work equally well. The key used to refer to genes was the gene locus ID.

The tables described in Figure 2 in Text S2 were used to generate large spreadsheets, which were analogous to complex database queries in a database system. The general format of these spreadsheets is shown in Figure 3a in Text S2. A specific example from one of the reconciliation sheets is also displayed in Figure 3b in Text S2 with an identical format as that shown in Figure 1a in Text S2 for comparison. This example is for reconciliation of the reaction maleylacetoacetate isomerase (MLACI), which has some gene associations in both iMO1056 and iJP829. The various fields in this worksheet are derived from the tables shown in Figure 2 in Text S2, primarily through use of the VLOOKUP function to pull in relevant data for each reaction and gene from the appropriate tables. Generally, one sheet used in the reconciliation process would include this organization of information for up to hundreds of reactions that were similar in their gene associations, with a different sheet for each category listed in Table 1 of the main text (or sometimes multiple sheets for a given category, if further subdivisions were warranted). All of the information relevant to a given reaction would be listed in a set of rows, below which would be a set of rows for the next reaction, etc.

Generating these sheets is not necessarily trivial or intuitive, so we have shown the specific methodology used to organize the information in Figure 4 in Text S2. This involved two indexing columns, one for the ‘setup’ index, and the other for the ‘functional’ index. By resorting by these two sets of indices, the reconciliation tables can be easily manipulated (as shown in Figure 4a in Text S2) or grouped with all relevant information for a given reaction clustered together for reconciliation (as shown in Figure 4b in Text S2). Reconciliation notes written in the center columns of these sheets are transferred to a separate reconciliation notes file at the end of each work session, as pictured in Figure 3c in Text S2. Once all reactions were annotated with reconciliation notes, these notes were used to make the necessary changes to the models, resulting in the final reconciled models. Full reconciliation notes for the reconciliation of *P. aeruginosa* and *P. putida* are listed in Table 5 in Text S1.

|  |
| --- |
| **Figure 3.** Layout of worksheets for analyzing reactions. **(a)** basic Excel layout of a worksheet for reconciliation of a given reaction. **(b)** specific example of layout for a given reaction. **(c)** sample of the ‘model reconciliation notes’ sheet, in which the decisions from the reconciliation process were recorded for each reaction. |

|  |
| --- |
| Figure 4. Technical setup of reconciliation sheets. The key to generating these excel sheets is the inclusion of two index columns, labeled ‘index1’ and ‘index2’ in the figure. **(a)** shows the figure sorted by index1, and **(b)** shows the figure sorted by index2. In **(a)**, various fields relevant to reconciling the reactions are separated from each other, and new fields can be added or new information added to fields easily for many reactions or genes at once. By resorting the table by index2, all of the fields for a given reaction group together, and each reaction to be reconciled (along with all of its auxiliary data) is separated by a bold orange row. This is a functional arrangement of data which can be used for reconciling reactions. |

# II. Categories of Changes made during reconciliation

Changes made to the metabolic GENREs of *P. aeruginosa* and *P. putida* as a result of the reconciliation were categorized into a number of categories that are grouped into four meta-classes, as shown in **Figure 2b** (main text) and described in Results (see Results section: “Assessing the impact of reconciliation on the reconstructions”). This section of the supplement describes the classes of changes, and gives some illustrative examples to explain how the classes relate to the biology of the GENREs.

*(i) “No change” meta-class*

The ’no change’ meta-class constitutes by far the largest group in either reconstruction. In approximately four-fifths of these conserved reactions the GPRs remained identical to the original reconstructions, while the remainder of the reactions required a modification of the GPRs to establish consistency between both reconstructions. These GPR changes have no direct influence on purely metabolic (i.e. flux) predictions, but they may influence analyses that incorporate modifications of the genetic status of the cell. For instance, if one of two putative isozymes was removed from a reaction during reconciliation, then the pre- and post- reconciliation GENREs would display different phenotypes in the case of an *in silico* knockout of the remaining gene. Therefore, these GPR changes are important for the accuracy of the reconstructions particularly in cases of single and multiple *in silico* gene knockouts.

*(ii) “Added” meta-class*

The ‘added’ meta-class is the second largest group of reaction changes. The majority of reactions in this meta-class were added to one reconstruction to account for functions that had been initially present only in the other reconstruction, but that turned out to be associated with reciprocally present genes. These additions were generally secondary functions or stoichiometries of certain enzymes, which might have been included in one organism but not in the other. In addition to reactions added for this reason, certain pathways or reactions were added that had been left out from one of the original reconstructions (e.g., fatty acid oxidation and some virulence pathways, which were initially reconstructed only in *P. putida* and *P. aeruginosa* respectively). Standardization of these processes between the two reconstructions generally meant adding reactions to the model for which these processes were not originally reconstructed, if the relevant genes were present.

*(iii) “Removed” meta-class*

Reactions removed from the respective reconstruction (the ‘removed’ meta-class) make up less than ten percent of the original reconstructions. Similarly to the ‘added’ meta-class, these functions were generally removed because a function or stiochiometry was not confirmed or was denied by the annotation of the reciprocal genes, or the re-evaluation of the available information contradicted the original decision. Furthermore, the model cleanup performed during the reconciliation process resulted in removal of several reactions from the reconstructions due to redundancy. This was the case, for instance, for some ‘lumped’ reactions whose stoichiometries were identical to the sum of several other reactions that were also present in one of the reconstructions.

*(iv) “Minor change’ meta-class*

The ‘minor change’ meta-class is the most disparate meta-class. It makes up approximately twelve and eight percent of the *P. aeruginosa* and *P. putida* reconstructions, respectively. This class represents reactions whose functions were preserved in the reconstructions, but for which some change was made in the implementation of the functions. These changes can be grouped into five types. The first is a change in reversibility. Since reaction reversibility is often based on vague or incomplete thermodynamic evidence, the reconciliation process involved aligning reversibility of reactions that were otherwise identical given the evidence available for both organisms. The second sub-class is ‘function neutral stoichiometry change.’ Many reactions involve cofactors or donors of different functional groups (e.g. amino group), yet it is often difficult to guess from the annotation which cofactor or donor is used by a particular enzyme. Therefore the most probable stoichiometry was adopted for both reconstructions in the case where the reactions were catalyzed by reciprocal genes. The ‘function reimplementation change’ subclass–the third type–contains reactions involved in metabolic functions that were realized differently in the initial reconstructions, despite similar or identical functions. An example of such a difference is ‘2-oxoglutarate dehydrogenase,’ which converts succinyl-CoA into 2-oxoglutarate. This reaction is catalyzed by an enzymatic complex and is a multi-step reaction, in that it produces a number of transient intermediates during the enzymatic process. Consequently, this enzymatic function can be represented in a reconstruction by either single (lumped) reaction or four reactions acting together, yet it still performs the same enzymatic function. As this function was implemented differently in the two initial reconstructions, appropriate changes needed to be made to avoid the existence of apparent (but non-functional) differences. The last two types, namely ‘reimplementation of organism-specific reactions’ and ‘reimplementation of full pathways,’ contain reactions that were modified based on new information pertaining either to the exact composition of compounds in the organisms or to the mechanisms of certain pathways that became available after the initial reconstructions were published. In this reconciliation, the ‘reimplementation of full pathways’ category included reconstruction of phospholipid synthesis pathways and the production of lipopolysaccharide (LPS). The synthesis of LPS was re-implemented completely based on a publication [1] that appeared after the original reconstruction process had been finished.

# III. Re-examination of the in silico LB medium

LB medium does not contain cysteine but rather cystine, a dimerized form of the amino acid [2]. For the initial validation of iMO1056, cysteine was not included in the *in silico* LB medium. However, we guessed that *P. aeruginosa* might be able to consume cystine either directly or through proteolysis of the amine bond. Therefore, to investigate the composition of the *in silico* LB medium, iMO1086 was grown *in silico* on rich medium with cysteine present. The inclusion of cysteine caused the improvement of the call of six genes to improve (FP→TN conversion) and none to worsen when compared to the analysis of the reconciled reconstruction using the original medium (see **Table 10 in** Text S1). The inclusion of three nucleotides (see Methods) caused further improvement of the calls for four genes (FP→TN) with a concomitant worsening of the call of a single gene (TP→FN), as shown in **Table 10 in Text S1**. These alterations to the *in silico* LB medium increased accuracy of iMO1086 to 85%, confirming that sources of purines and pyrimidines as well as L-cysteine were likely present in the LB medium used for the genome-wide transposon studies, and thus should be included in the *in silico* rich medium.

# IV. Analysis of changes in yields

In order to assess how the reconciliation process affected the yield predictions of the reconstructions, flux balance analysis (FBA) simulations were performed for growth of the original and the reconciled reconstructions. The *in silico* maximal yield determined on glucose minimal medium was used as a metric to describe efficiency of the metabolic networks. To compute *in silico* maximum yield, FBA simulations were performed with the Non-Growth-Associated Maintenance (NGAM) parameter set to zero (thus allowing all carbon uptake to be channeled into biomass production) and the Growth-Associated Maintenance (GAM) set to the same value as in the respective original reconstruction (GAM is modeled as a hydrolysis of ATP as part of the biomass equation) [3]. For both the *P. putida* and *P. aeruginosa* reconstructions, maximal yield increased as a result of the reconciliation (see **Table 7 in Text S1**). In the *P. putida* reconstruction this increase was two and a half times as large (9.4%) as that seen in the *P. aeruginosa* reconstruction (3.6%), an increase that indicates that more efficiency was gained via reconciliation-derived changes in the *P. putida* model than in *P. aeruginosa*. This increase in yield can be explained partly by an increase in efficiency of the oxidative phosphorylation pathway from the reconciliation process. Specifically, the *in silico* P:O ratio increased in both reconstructions from 1.5 to 1.875. In the *P. aeruginosa* reconstruction, the increase in maximal yield can be completely explained by the increased P:O ratio. In fact, removing the effects of changes in the P:O ratio, the maximal yield in the reconciled *P. aeruginosa* reconstruction is actually slightly lower (by 3%) than that of the original reconstruction, a decrease in yield that was partially (one third) caused by the change in the stoichiometry of the 2-ketogluconate transporter (exchanging simple diffusion with proton symport), through which the uptake of glucose (after converting it extracellularly to gluconate) proceeded. In the *P. putida* reconstruction the increase of P:O ratio was responsible for around three-fourths of the increase in yield (see **Table 7 in Text S1**). It is worth noting that while both *P. aeruginosa* reconstructions use the same biomass composition, the reconciled *P. putida* reconstruction uses a slightly modified biomass composition that was experimentally determined (Puchalka et al, in preparation). Reversion of the *P. putida* reconstruction to the original (pre-reconciliation) biomass composition and P:O ratio brings the yield of the reconciled GENRE to within 0.5% of the yield of the original reconstruction, indicating that these two factors dominate the changes observed in yield. Even together with the increase in P:O ratio, however, the changes to maximal yields were small when compared to the accuracy of feasible experiments for determining yield.

# V. References

1. King J, Kocincova D, Westman E, Lam J (2009) Review: Lipopolysaccharide biosynthesis in Pseudomonas aeruginosa. Journal of Endotoxin Research 15: 261.

2. Oh YK, Palsson BO, Park SM, Schilling CH, Mahadevan R (2007) Genome-scale reconstruction of metabolic network in Bacillus subtilis based on high-throughput phenotyping and gene essentiality data. J Biol Chem 282: 28791-28799.

3. Varma A, Palsson BO (1994) Stoichiometric Flux Balance Models Quantitatively Predict Growth and Metabolic by-Product Secretion in Wild-Type Escherichia-Coli W3110. Applied and Environmental Microbiology 60: 3724-3731.
